# Learning analytics dashboard: a tool for providing actionable insights to learners

**DOI:** 10.1186/s41239-021-00313-7

**Published:** 2022-02-14

**Authors:** Teo Susnjak, Gomathy Suganya Ramaswami, Anuradha Mathrani

**Affiliations:** School of Mathematical and Computational Sciences, Massey University, Auckland, New Zealand

**Keywords:** Dashboard, Learner analytics, Actionable insights, Model interpretability, Explainable AI, Counterfactuals

## Abstract

This study investigates current approaches to learning analytics (LA) dashboarding while highlighting challenges faced by education providers in their operationalization. We analyze recent dashboards for their ability to provide actionable insights which promote informed responses by learners in making adjustments to their learning habits. Our study finds that most LA dashboards merely employ surface-level descriptive analytics, while only few go beyond and use predictive analytics. In response to the identified gaps in recently published dashboards, we propose a state-of-the-art dashboard that not only leverages descriptive analytics components, but also integrates machine learning in a way that enables both predictive and prescriptive analytics. We demonstrate how emerging analytics tools can be used in order to enable learners to adequately interpret the predictive model behavior, and more specifically to understand how a predictive model arrives at a given prediction. We highlight how these capabilities build trust and satisfy emerging regulatory requirements surrounding predictive analytics. Additionally, we show how data-driven prescriptive analytics can be deployed within dashboards in order to provide concrete advice to the learners, and thereby increase the likelihood of triggering behavioral changes. Our proposed dashboard is the first of its kind in terms of breadth of analytics that it integrates, and is currently deployed for trials at a higher education institution.

## Introduction

Analytics technologies have proliferated across many sectors of society for extracting data-driven insights, improving decision making and driving innovation. The tertiary educational sector is particularly in-tune with the advantages that data analytics can offer, and generally, seeks to leverage these advances. Deployment of analytics technologies is becoming increasingly important as this sector is undergoing disruptions across different parts of the world, as well as due to the COVID-19 pandemic crisis (Aristovnik et al., 2020). The current pandemic responses have shifted education delivery to online modes, further accelerating ongoing disruptions. The education sector is already facing financial and competitive pressures (Muhammad et al., 2020) in some regions, and this global shift to online learning has amplified them even more. These shifts have altered the competitive landscape between universities in countries with low or non-existent subsidies with those located in countries that have strong government support by bringing them into direct competition as geographic and physical boundaries now have a diminished relevance. This has been accentuated by the continuing rise in higher education costs (Blankenberger & Williams, 2020), together with the questioning of the value proposition that higher education offers in many of the available qualifications. These challenges call for a need to adapt and do things differently. For these reasons, data analytics with its use of innovative products has risen to become one of the key enablers in the educational sector. Analytics in this sector focuses on maximizing student retention rates by identifying the at-risk students in the early stages and then by initiating interventions, whilst improving the quality of the educational experience for the learners.

One of the analytics innovation products that have been deployed to benefit the enrolled learners, are Learning Analytics dashboards (LADs) which have been operationalized in numerous institutions (Table 1) in the last few years. The purpose of existing LADs is not dissimilar to the dashboards widely used in industry. They aim to provide learners with a snapshot of how they are progressing in their courses. Graphical displays highlight trends in the learners’ academic and engagement levels through the digital footprints generated by learners and provide them with a basis for awareness, reflection and new insights (Yoo & Jin, 2020). In more advanced cases, the dashboards are designed to make predictions on where learners are likely to end up in respect to meeting learning outcomes based on their current trajectory. Analytics tools reveal learner insights that could prompt a reflection process that would otherwise not be realized. It is assumed that these reflections may (in some cases) trigger positive behavioral changes that support learners in maximizing learning outcomes and course completions as well as retention rates.

**Table 1:** Reviewed papers overview

Analysis		Studies
Descriptive analytics content	Conducted	Aljohani et al., 2019; Baneres et al., 2019; Bodily et al., 2018; Chatti et al., 2020; Chen et al., 2019; Fleur et al., 2020; Gras et al., 2020; Han et al., 2021; He et al., 2019; Karaoglan Yilmaz & Yilmaz, 2020; Kia et al., 2020; Kokoç & Altun, 2021; Majumdar et al., 2019; Naranjo et al., 2019; Owatari et al., 2020; Ulfa et al., 2019; Valle et al., 2021
Predictive analytics content and reported accuracy	Not conducted	Aljohani et al., 2019; Bodily et al., 2018; Chatti et al., 2020; Chen et al., 2019; Gras et al., 2020; Han et al., 2021; He et al., 2019; Karaoglan Yilmaz & Yilmaz, 2020; Kia et al., 2020; Majumdar et al., 2019; Naranjo et al., 2019; Owatari et al., 2020; Ulfa et al., 2019
	Conducted	Baneres et al., 2019; Fleur et al., 2020; Kokoç & Altun, 2021; Valle et al., 2021
	Accuracy not reported	Fleur et al., 2020; Valle et al., 2021
	80–89% accuracy achieved	Kokoç & Altun, 2021
	90–95% accuracy achieved	Baneres et al., 2019
Prescriptive analytics content	Conducted	None
	Conducted non-data driven	Baneres et al., 2019; Bodily et al., 2018; Gras et al., 2020; Han et al., 2021; Karaoglan Yilmaz & Yilmaz, 2020; Majumdar et al., 2019
	Not conducted	Aljohani et al., 2019; Chatti et al., 2020; Chen et al., 2019; Fleur et al., 2020; He et al., 2019; Kia et al., 2020; Kokoç & Altun, 2021; Naranjo et al., 2019; Owatari et al., 2020; Ulfa et al., 2019; Valle et al., 2021
Model interpretability and explainablility	Conducted	None
Dashboard evaluation and effectiveness	Evaluation conducted within a pilot study context	Bodily et al., 2018; Chatti et al., 2020; Gras et al., 2020; Han et al., 2021; He et al., 2019; Kia et al., 2020; Kokoç & Altun, 2021; Naranjo et al., 2019; Owatari et al., 2020; Ulfa et al., 2019
	No evaluation conducted within a prototype study context	Chen et al., 2019; Majumdar et al., 2019
	Positive effects on student outcomes reported	Aljohani et al., 2019; Fleur et al., 2020; Han et al., 2021; Kokoç & Altun, 2021
Dashboard Color content	1-3 colors	Fleur et al., 2020
	4-6 colors	Bodily et al., 2018; Kia et al., 2020; Naranjo et al., 2019; Ulfa et al., 2019; Valle et al., 2021
	> 6 colors	Aljohani et al., 2019; Baneres et al., 2019; Chatti et al., 2020; Chen et al., 2019; Gras et al., 2020; Han et al., 2021; He et al., 2019; Karaoglan Yilmaz & Yilmaz, 2020; Kokoç & Altun, 2021; Majumdar et al., 2019; Owatari et al., 2020)

However, operationalizing these types of analytics products is accompanied with numerous challenges. Given the financial investment and the human resource effort involved in their productionization (Mahroeian et al., 2017), it is also not altogether clear what visual elements LADs should possess, that is, what type of information is effective at triggering positive behavioral adjustments in learners, or what aspects are detrimental by potentially inducing anxiety among learners. Overall, evidence about whether learning analytics and LADs improve learning in practice is scarce (Ferguson & Clow, 2017; Guzmán-Valenzuela et al., 2021; Knight et al., 2020; Rets et al., 2021; Wilson et al., 2017) and has room for further investigation.

This paper expands on the challenges of operationalizing LADs and identifies gaps in current LADs. It does this by integrating analyses of recently published LADs by following a systematic line of inquiry. The following subsections offer definitions of various analytics layers that we use in our analysis of the current state of practice in LADs, as well as an outline of our research agenda for establishing an institutional-level dashboard that offers more value to learners.

### Analytics layers

Analytics can provide different levels of informational insights to enable users in making informed decisions. At the most basic level, descriptive analytics highlight snapshots of variables of interest. These convey information about trends and the current status relative to other identified measures. Descriptive analytics are the simplest form of insights to extract from data, and while useful, have a limited utility.

Predictive analytics on the other hand emphasize some form of forecasting and embody the ability to estimate future outcomes based on current and past data patterns. Predictive analytics are mostly driven by machine learning algorithms which learn from historic datasets in order to produce classifiers that can make inferences about possible future outcomes from current data inputs. Data products based on predictive analytics represent a considerable increase in complexity over mere descriptive analytics and offer more value, but they also possess shortcomings. One shortcoming is that they usually produce black-box models which lack transparency into their internal workings (Adadi & Berrada, 2018). This means that it is often not possible for users to understand how these models make predictions, and what aspects of the learners’ behaviors are driving the predictions towards prognosticated outcomes. This lack of model *interpretability* and *explainability* of outputs associated with most predictive models lowers their utility, and over time erodes the trust of users (Baneres et al., 2021). Therefore, a trend is emerging at regulatory^1^ levels requiring predictive models to expose their reasoning behind the predictions in comprehensible ways.

The most complex and arguably the most insight-rich form of analytics is prescriptive analytics. Prescriptive analytics can leverage predictive analytics in such a way that the underlying models are also able to infer possible causal relationships and consequently generate recommendations and suggestions to users about which specific behavioral changes are most likely to result in positive outcomes. These prescriptive outputs are tailored to each learner, but their suggestions are *data-driven* and thus based on similar students who achieved positive outcomes in the past. By issuing advice on behavioral adjustments and learning strategies that learners can undertake to maximize their learning outcomes, the decision-making process for the learners can be simplified and the guesswork removed.

Currently, descriptive LADs are most commonly in use with an increasing number integrating predictive components. However, to the best of our knowledge, examples of dashboards incorporating data-driven prescriptive aspects of analytics do not exist.

### Aims

This paper has three parts and contributions. We first provide an overview of the existing challenges in developing institutional LADs. We highlight three challenges (namely, representation and actions, ethics, and agility) faced in deploying LAD initiatives involving student-facing dashboards. Secondly, we provide an extensive survey of the most recently published state-of-the-art LADs in literature. Our search identified 17 LADs and we assess their common characteristics as well as strengths and weaknesses. Thirdly, we propose our LAD which addresses many of the shortcomings, and to the best of our knowledge, is the first LAD that brings descriptive, predictive and data-driven prescriptive analytics into one display. We conclude with offering inferences on what we see as being future directions and emerging frontiers in LA dashboarding in the short to medium term.

Our research questions are as follows:

1. What are unique challenges in developing student-facing LADs?
2. How ubiquitous are LADs?
3. What is the current evidence for the effectiveness of LADs?
4. What are the strengths and weaknesses of the current approaches to LA dashboarding?
5. What are future directions of LA dashboarding and how can some of the existing weakness be addressed?

## Analytics in education

There are broadly three streams of research within educational analytics. Learning Analytics (LA) focuses on learners. Its primary concern is optimizing teaching and learning processes. Educational Data Mining (EDM) on the other hand seeks to develop methods for exploring educational data in order to better understand learners, and to extract insights about them and the educational systems. Academic Analytics (AA) draws on the insights gained from educational data for supporting strategic decision-making, resolving academic issues such as retention and improving marketing strategies.

These three streams intersect at various points and share much of the underlying data, and though they could all be grouped under the same umbrella as Educational Data Science, they differ in the stakeholders which they target. EDM tends to target both teachers and learners, while LA primarily addresses the needs of learners. However, institutional administrators, managers and educational policymakers are the key stakeholders of AA applications. The three streams also affect different levels of the educational systems. LA is linked to course-level granularity and to department-level concerns within institutions, while EDM spans departmental through to faculty and institutional-level concerns (Nguyen et al., 2020). Meanwhile, AA affects universities at the institutional level, that has implications for policy making, thus it spans regional, national and possibly international levels.

### Challenges for building LADs

While there are some differences between LA, AA and EDM, they all share some common challenges. Numerous studies have reported implementation details of LA products; however, a recent study by Leitner et al. (2020) pointed out that they rarely provide comprehensive descriptions of challenges faced in productionizing these systems. This study shortlisted seven general challenges for deploying LA initiatives:

1. Purpose and Gain: managing expectations of different stakeholders.
2. Representation and Actions: facilitation of actionable insights by LA products.
3. Data: communication to students regarding what is being done with their data, and formulating suitable policies to manage data processes.
4. IT Infrastructure: balancing the pros and cons of opting to use internal or external service providers for implementing and running the LA products.
5. Development and Operation: planning and implementation of the process of developing and operating an LA initiative.
6. Privacy: ensuring both security of learners’ data and compliance with increasingly stringent legal requirements worldwide.
7. Ethics: ensuring that LA products do not bring harm and provide learners with the ability to opt-out.

The above challenges are generic and broadly applicable to all LA projects. We draw on recent literature to expand on two particular challenges above (2 and 7), and we tailor them to the difficulties which specifically relate to LAD projects. In addition, with supporting literature we posit an additional challenge, namely Agility, to the original seven identified by Leitner et al. (2020).

### Representation and actions

Dashboard visualization is more of a science than art. The dashboard designer must possess a degree of understanding of how the human visual cortex perceives various visual cues in order to optimally match different data types to suitable visual representations. Some data are quantitative and others are ordinal or categorical in their attributes. The values of each data type are best represented by different cues which could comprise contrasting colors, differing spatial positions or variations in symbols denoting length, size, shape and orientation amongst others. The designer also needs to possess both domain expertise in learning theories and paradigms, as well as technical capabilities in developing dashboards (Klerkx et al., 2017).

Choosing the correct visualization technique can present difficulties largely due to the increasing amounts of available data and the candidate variables/indicators that can be incorporated (Leitner et al., 2019). Ensuring that dashboards are informative without overwhelming the user is a challenging balancing act. From an aesthetic perspective, Tufte (2001) cautions against use of ‘non-data-ink' and ‘chartjunk’ in graphs, that is, he maintains that excessive use of colors, patterns or gridlines can confuse and clog the recipient's comprehension. Bera (2016) specifically mentions the overuse and misuse of color in business dashboards and the role this has on the users’ decision-making abilities. Bera’s research finds that contrasting colors vie for user’s attention, and unless necessary, they distract and affect the decision-making processes. By using eye tracking technology, the study demonstrated that the cognitive overload associated with the misuse of color in dashboards leads to longer fixation periods on irrelevant aspects of dashboards and prolongs the ability of users to comprehend the information.

Use of predictive modelling is becoming more prominent within LA (Bergner, 2017), and these techniques are emerging more frequently within dashboards. A recent study (Baneres et al., 2021) into developing LA technologies acting as early warning systems for identifying at-risk learners highlighted the need to move beyond ‘old-fashioned’ dashboards that simply rely on descriptive analytics and to instead, orient efforts towards incorporating predictive analytics amongst other advanced features. However, building highly accurate and reliable predictive models is not trivial. Firstly, it requires considerable technical expertise which is not always easy to acquire. Secondly, predicting outcomes based on human behavior reflects a non-deterministic problem. Further, for scalability reasons, we ideally require generic predictive models which can predict student outcomes across widely disparate courses. However, since courses have different attributes, styles of delivery and assessment types, it is a considerable challenge to create single generic predictive models that can work optimally across diverse courses. On the other hand, developing tailored predictive models for each different course creates technical resource overheads. Tailored models are also likely to perform badly in many instances due to scarcity of data leading to overfitting, since individual courses may have small class numbers or have limited historical data. A recent systematic literature review on the current state of prediction of student performance within LA, Namoun and Alshanqiti (2020) found that the state of predictive modeling of student outcomes is not fully exploited and warrants further work. The study found that not only the accuracy of the existing models has room for improvement, but more robust testing for its validity, portability (or generic models) and overall generalizability needs to be conducted. In a recent study, Umer et al. (2021) concluded that many datasets used to build predictive models in this domain were small, often having less than 10% of the overall data points for certain class labels, leading to unreliable predictive accuracies especially when course-tailored predictive models are being created. The study also calls for enhancing the scope of engagement data to cover learner interaction data from forum messages, identifying pedagogically meaningful features and developing dashboard visualizations that have some underlying pedagogical intent.

Developing accurate classifiers is further complicated by the negative effects of concept drift (Lu et al., 2018). Concept drift describes the degradation in accuracies of predictive models over time since data used to build models may become disconnected with current real-life data. This can occur when learners’ study patterns gradually or abruptly change (as in the case of pandemic responses), and current digital footprints no longer correlate with previous patterns in the historic record. For example, the gradual shift towards the use of virtual learning environments (VLE) over the last 10–15 years represents a concept drift. Learners’ study patterns prior to this period in the historic record bear little resemblance to the patterns of learners of today, and thus, data from historic period will likely degrade predictive accuracies of current students. Concept drift can also happen suddenly, as indeed the sudden migration to full online learning during the recent pandemic crisis brought into play additional technologies and different digital patterns and footprints that students leave behind. This disconnect between the independent and dependent variables from historic data needed to train the predictive models, with the independent variables being used as input to predict the outcomes of current students, is constantly evolving. This phenomenon represents a technical and a capability challenge for universities, as concept drift needs to be detected and accounted for, while the mechanisms for achieving this effectively are still being researched (Lu et al., 2018).

The above challenges are considerable. However, even if they can all be addressed, it is now no longer sufficient to deploy predictive models and solely display their outputs without providing the learners with explainability of how a model arrived at a given prediction. It is also becoming more apparent that learners will engage with a LAD only if they understand how displayed values are generated (Rets et al., 2021). Liu and Koedinger (2017) argue for the importance of interpretability which leads onto actionability. Models need to possess explanatory characteristics so that learners understand why a model produced given predictions, what the underlying driving factors are, and importantly, what insights can be derived from these explanations in order to trigger actionable behavioral adjustments. Not only should interpretability of models and explainability of their individual predictions be provided to the learners, but also counterfactuals, which explicitly demonstrate alternative outcomes for the learner if a behavioral change were to take place in specific areas. Recent studies (Rets et al., 2021; Valle et al., 2021) in LADs have highlighted the necessity of integrating insights which are prescriptive and take on forms of recommendations to guide students in their learning. Producing such rich and sophisticated outputs is a challenge, because extracting simplified representations of predictive black-box models and their reasoning is complex. There are limited available tools with sufficient maturity that support this functionality, which again requires a high level of expertise to implement and leverage.

### Ethics

The challenges surrounding ethical use of data within LA products are generally well understood and accepted. They center around questions of what personal data should be collected and processed by these systems, what insights should be extracted and with whom they should be shared. Additional concerns exist around possible consequences on learners when conveying personalized information; therefore, institutions need to be aware of intrusive advising or inappropriate labelling that may lead to learner resentment or demotivation (Campbell et al., 2007). As such, avoidance of harm to learners, alongside compliance with legal requirements are paramount.

Given the importance of practical ethical underpinnings when using LA systems, it is acknowledged that robust and clear policies need to be formulated on what empirical data is permitted to be used for analytical purposes and to what end (Kitto & Knight, 2019). The study supports that awareness of these policies must be communicated to the learners together with the purported educational benefits that such systems claim to bring, together with the potential risks. A key concern however is the uncertainty regarding the benefits distribution, which may not be the same for everyone (Rubel & Jones, 2016); hence, institutions are encouraged to create a sense of transparency about LA systems by including statements on their data practices and limitations.

Beyond the well accepted dilemmas of LA systems listed above, predictive models used in LADs bring with them some other acute challenges. Predictive models naturally embody within them the process of generalization. As the machine learning algorithms learn and induce predictive models, they move from individual and specific examples to more general descriptors of the data. With this natural induction process, errors are invariably introduced. The ethical concern and challenge come into play when we consider both incorrect and correct classifications and the effects that they might have on learners. If a student is mis-classified as being “at-risk” this might have the effect of discouraging them and eventuate in the “fulfillment of the prophecy” despite the fact they were originally on-track to successful completions. Or, in using oversimplified classification labels, we can diminish the predictive value and in turn reduce the trustworthiness of the analytical approach. This challenge will always remain since learners are not deterministic and predictive models in non-deterministic domains are inherently imperfect. Likewise, Bowker and Star (2000) note that even with correct predictions, for some this may be an incentive if they are already motivated and capable of positively adjusting their course in order to alter their predicted outcome, while for others, the prediction may only serve to further deflate.

### Agility

Agility is the ability to rapidly adapt to changing requirements, be flexible and able to seize new opportunities. Universities are more resistant to change than industrial entities (Menon & Suresh, 2020); they are typically considered to be fractured and decentralized (Bunton, 2017), while possessing complex and non-standard business processes (Mukerjee, 2014a). However, financial constraints coupled with pressure from competition as a consequence of the unfolding digital revolution, have put universities on high alert to engage with new technologies (Mukerjee, 2014a). It is recognized that organizational agility is a crucial capability for universities at these times (Mukerjee, 2014b). Both the use of data insights and analytics as well as the development of these projects, places immediate demands of agility on behalf of the organization operationalizing them. Agility is therefore a key challenge for universities attempting to productionize LADs.

The requirement for agility comes at different levels in respect to LADs. Translating LADs into products that genuinely improve learning outcomes requires constant monitoring and analysis of their usage patterns, user feedback and ultimately the gathering of evidence into their efficacy. The consequences of this are an increase in resource costs for maintenance and continuous refinement of the LADs. Continuing support from the institutions and willingness to provide ongoing long-term refinements need to be secured ahead of time. Sun et al. (2019) point out that improvements of these types of systems needs to go beyond pilot and deployment stages, and that underlaying assumptions used to develop these systems need to be re-assessed as adjustments are made to enhance the design or functionality. For best results, the design of dashboards should be iterative with continuous feedback from learners in order to ensure that an operationalized product is actually useful. This is time and resource intensive and requires agility.

From a data-oriented point of view, agility and the ability to integrate new data streams into LADs are paramount. Universities are rapidly incorporating modern technologies for course delivery and improving the learning experience. The technologies sometimes augment what is already in place, while other times, they completely replace legacy processes and systems with new ones. This process has been accelerating recently and will continue to do so. The consequence is that new and more diverse digital footprints will continue to be generated by learners especially with the increased demand in online education in the aftermath of COVID-19. Therefore, adaptability and rapid responses in integrating new data sources must be set forth to identify new features that can improve the predictive power of deployed models.

Finally, profound insights are compelling. They demand action if negligence is to be avoided. Deep insights can be game-changers and often call for swift action even when this is inconvenient. For example, if predictive models powering the LADs identify certain qualifications within an institution’s portfolio as being key predictive drivers towards poor completion rates, then this would need to trigger action and possibly advice on changes that may neither be convenient for an institution, nor even align with their overarching strategic goals. With deployment of LADs, therefore, comes the responsibility of asking the tough questions in adapting to the suggested changes that can have a better institutional impact.

## Methods

The focus of this study was to review the most recent developments in LADs. To that end, the search focused on studies published from 2018 until the time the search was completed (September 2021). The search followed the PRISMA framework (Moher et al., 2009) which requires a principled approach to defining the inclusion and exclusion criteria as well as search parameters.

We first conducted a keyword search targeting Google Scholar using the “Publish or Perish” tool in order to retrieve the initial academic articles. The following search terms were used: “learning analytics dashboard” or “visualization tool” or “early warning system” or “student dashboard”. The search yielded the following total number of results per year: 2018 n = 340, 2019 n = 977, 2020 n = 960, and 2021 n = 403. A total of 2680 papers were obtained. This was reduced to 1450 papers following the elimination of duplicates.

**Fig. 1:**
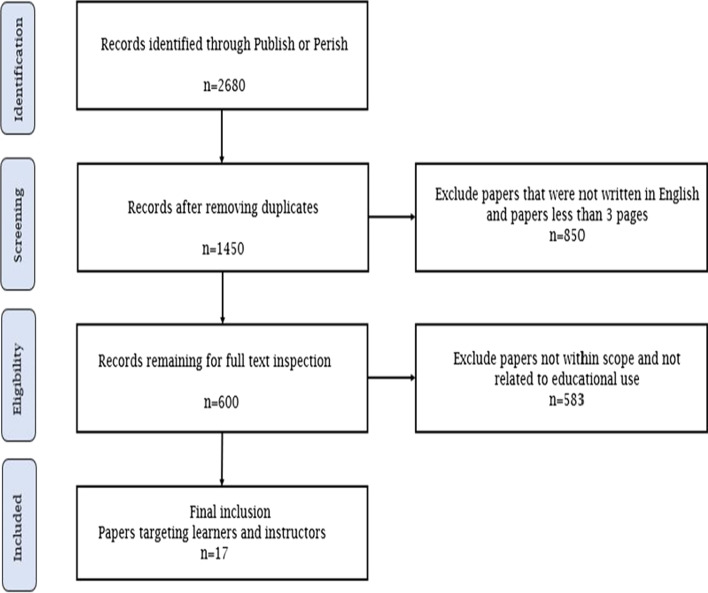
Methodology used in this systematic review (Moher et al., 2009)

**Fig. 2:**
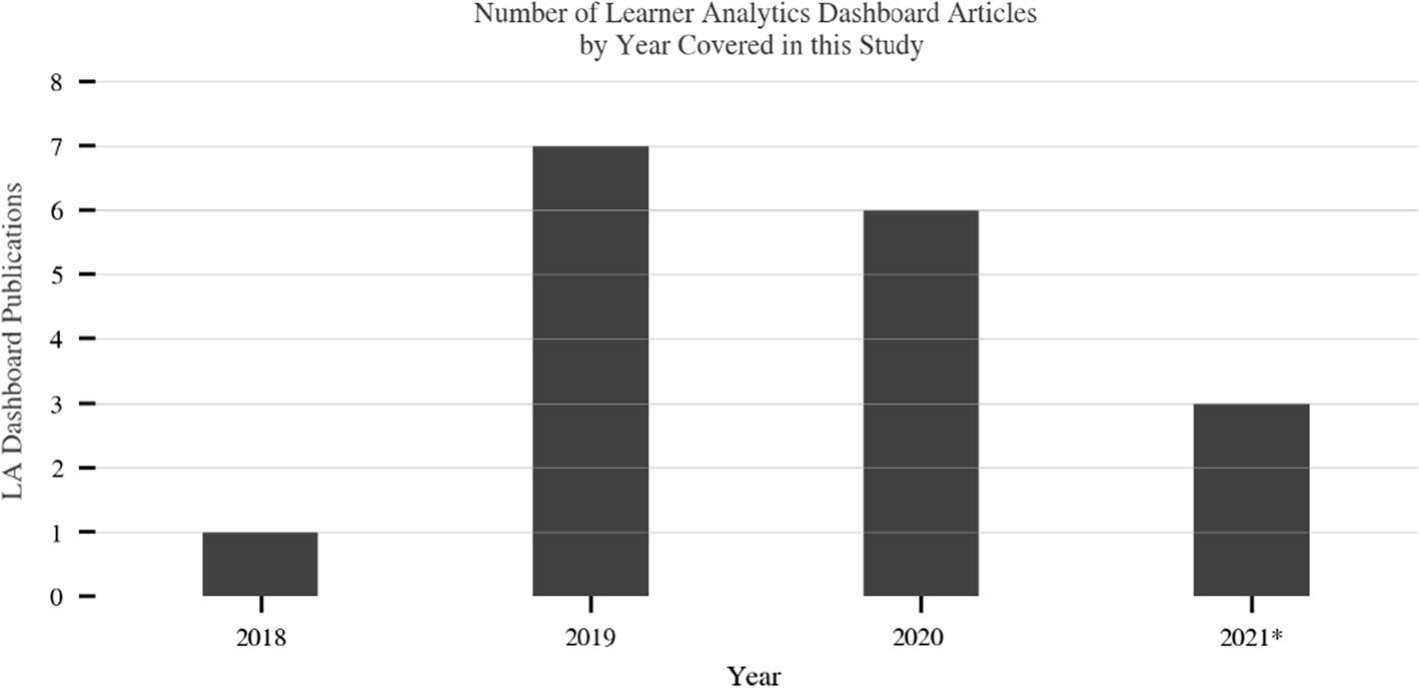
Total number of published articles presenting LADs that are covered in this study. The number of publications for 2021 is listed up to September of that year.

Next, papers that were not written in English and those containing less than 3 pages were filtered, resulting in 600 papers. The abstracts of these papers were screened, and finally only the papers that focused on dashboards targeting learners and instructors were retained. This yielded a total of 17 papers that successfully passed all the inclusion criteria and only these were included in the final analysis. Figure 1 outlines the overall methodology used for data collection, while Fig. 2 depicts the histogram of the 17 LAD papers by year.

Against the backdrop of recently published literature, the first part of our study has already identified challenges facing LA and specifically difficulties associated with the development and deployment of dashboards in educational contexts. The second part of our study analyzes the data on existing LADs. Our analysis approach is based on five key assertions that are grounded in dashboard literature. The assertions act as a prism through which we reviewed the LADs and directed our investigation towards the design of our dashboard subsequently. These are as follows:

1. Given that LADs using only descriptive analytics is not enough, it is meaningful to identify dashboards which have started to incorporate predictive and data-driven prescriptive analytics.
2. Since accuracy of predictive models is an identified challenge, it is informative to determine what accuracies are being reported for recent LADs using predictive modeling, and if they communicate the confidence of their predictive outputs to the learners.
3. Assuming that there is value in providing learners with some level of interpretability of the underlying predictive models and explanations of how the models have arrived at predictions for individual students, it is instructive for future research directions to ascertain how prevalent is the presentation of these features on the LADs.
4. Since the evidence of the effectiveness of LADs to affect positive outcomes for learners is not complete, it is instructive to know what evaluation efforts have been made in recent studies on the utility of LADs.
5. LADs using higher number of colors are more likely to misuse and overuse colors and contribute towards confusion for the learners.

## Analysis

Our analysis is divided into two parts. The first part reviews each of the 17 LADs and highlights noteworthy aspects of each one. The second part analyzes the dashboards at an aggregate level and offers analyses of the state of LA dashboarding in a summative format.

### Review of dashboards from literature

Our framework for reviewing all the LAD studies uses a scheme whereby we consider each LAD from the perspective of how they have implemented descriptive, predictive and analytics functionalities, as well as the reported evidence outlining the effectiveness of LADs on learner outcomes.

#### LADs with descriptive analytics capabilities

All studies incorporated some aspects of descriptive analytics. Frequently, the descriptive analytics were in the form of graphical displays depicting comparisons of a student in respect to class averages or patterns in relation to students (Aljohani et al., 2019) across metrics like assessment scores, participation levels and interaction with online activities (Chen et al., 2019; Fleur et al., 2020; Gras et al., 2020; Han et al., 2021; Karaoglan Yilmaz & Yilmaz, 2020; Kokoç & Altun, 2021; Ulfa et al., 2019; Valle et al., 2021). Some studies focused on status updates of progression through online course materials such as video, reports on time spent on eBooks and summaries of course notes (Bodily et al., 2018; He et al., 2019; Majumdar et al., 2019; Owatari et al., 2020). Other studies also assisted learners in planning and provided alerts of upcoming assessment submission deadlines (Baneres et al., 2019; Kia et al., 2020; Naranjo et al., 2019). Certain LADs (Chatti et al., 2020) went beyond static dashboards and enabled direct customizations of them by allowing learners to dynamically generate indicators of their choice.

#### LADs with predictive analytics capabilities

Several studies went further than mere descriptive analytics and incorporated predictive analytics elements into their dashboards. A descriptive and a predictive dashboard was developed by Valle et al. (2021). The descriptive dashboard aimed at displaying the students’ performance relative to the class average while the predictive dashboard displayed the probability of learners attaining specific grades. The authors reported that the predictive dashboard helped only the highly motivated students to sustain their motivation levels, while both dashboards failed to demonstrate their effectiveness in affecting final outcomes. Similarly, Fleur et al. (2020) developed a LAD with class-comparative descriptive components as well as the student’s predicted final grade. The students in the treatment group accessed the dashboard and their performance was analyzed in formative and summative assessments. The study reported that students in the treatment group performed better in the formative assessment only. Baneres et.al (2019) focused on devising an early warning system for learners and instructors that identifies at-risk students. Their Graduate At-Risk (GAR) model used grades to predict course outcomes. Additionally, an intervention mechanism was incorporated that automated sending personalized messages to at-risk students. While GAR noted an improvement in performance of the at-risk students, it could not be determined which factors were responsible. In a similar vein, a prescriptive learning dashboard (PLD) using personalized recommendation texts was developed by Kokoc and Altun (2021) which also focused on generating of student risk status and displaying it on the dashboard. The authors concluded that those students who used PLD performed significantly better in their courses. None of the examined studies took steps to communicate to students through the dashboard how reliable the underlying predictive models were, nor were technologies used which could elucidate to learners how the models operated, or how the predictions were generated based on specific student’s data.

#### LADs with prescriptive analytics capabilities

Certain studies already mentioned above like Kokoc and Altun (2021) and Baneres et at. (2019), considered the dispatch of personalized messages as prescriptive components. Indeed, a number of other studies also leveraged different forms of messaging, recommendation techniques and communication features through the LADs in order to claim prescriptive capabilities. Bodily et al. (2018) developed LADs that recommended content and skill-building activities such as practice exercises. Their study noted that skill-related recommendation components were found by students to be more useful compared to the content recommender features. Along similar lines, Karaoglan Yilmaz and Yilmaz (2020) took the approach of delivering weekly reports over the course duration along with personalized recommendations to each student. The study claimed that providing analytics reports positively increased student motivation. Gras et al. (2020) expanded the capabilities of their LAD by providing students with an action button which accessed direct help from the instructor and can therefore be categorized as having prescriptive aspects. Direct contact between students and instructors was also enabled by the face-to-face collaborative argumentation (FCA) dashboard developed by Han et al. (2021). This tool monitored students’ learning progress and facilitated prescriptive interventions by instructors with students requiring additional assistance. Meanwhile, LAView a dashboard was developed by Majumdar et al. (2019) which computed an engagement score as an aggregate value across several student interaction measures. Based on the engagement score, the instructor initiated prescriptive measures in the form of personalized emails to corresponding students. LADs are clearly emerging with some forms of prescriptive components, though many of these can also be defined as human interventions. Others which have more of an automated algorithmic approach to dispensing recommendations of content and activities are based on simplistic hard-code heuristics and thresholds. More sophisticated prescriptive components within LADs leveraging algorithmic and *data-driven* analytics have yet to emerge.

#### Reported effectiveness of LADs

Value assessments of the various LAD projects have taken two distinct approaches amongst the examined studies. Some studies (e.g., Bodily et al., 2018; Chatti et al., 2020; Gras et al., 2020; Han et al., 2021; He et al., 2019; Kia et al., 2020; Kokoç & Altun, 2021; Naranjo et al., 2019; Owatari et al., 2020; Ulfa et al., 2019) have largely conducted qualitative evaluations of the various LAD deployments within pilot contexts. These included surveys and interviews of usability aspects and covered subjective responses on the degree that LADs facilitated learning. However, other studies provided quantitative findings supported by statistical analyses that demonstrated that LAD usage had positive effects on student outcomes (Aljohani et al., 2019; Fleur et al., 2020; Han et al., 2021; Karaoglan Yilmaz & Yilmaz, 2020; Kokoç & Altun, 2021).

### Dashboard data analysis

Table 1 summarizes all the revised dashboards through the analysis approach listed in “Methods” Section. We find that 59% LADs include some form of descriptive analytics information to the learners. The remaining LADs focus on assisting students with planning, helping them monitor progress through online learning materials and provide learners with a medium through which instructors can more effectively interact with the learners.

The majority of the LADs either did not use any form of predictive analytics or did not report on this capability if implemented. This large group consisted of 76% of the most recently developed dashboards for learner-facing educational contexts. Out of the remaining 24% which did use predictive analytics, one of the dashboards generated predictive models with an accuracy range between 80 and 89%, while another achieved higher accuracies reaching up to 95%; however, while these accuracies were reported in literature, the model accuracies were not presented to the students on the dashboards. The remaining dashboards that used predictive analytics did not report on their predictive accuracies.

The data also indicates that model transparency approaches and technologies have not entered usage amongst the dashboard developers. Of all the dashboards which used predictive modeling, we find that no attempt was made to offer model interpretability to the learners in terms of what were the key features. Additionally, we find that the none of the reviewed dashboards tried to explain to the learners how the predictive models actually arrived at the predictions that were presented to them.

From our review, we found that none of the recent LADs utilize data-driven prescriptive analytics. Our data indicates that 47% used some form of prescriptive features associated with the dashboards which took the form of encouraging messages, supportive emails or instructive suggestions being issued to learners by the teachers. However, none employed automated instructions or recommendations generated by prescriptive modelling algorithms.

Widely contrasting approaches were adopted by researchers in respect to evaluating the usability of the dashboards, as well as their overall ability to affect positive learning outcomes. We found that 59% of the dashboards which were deployed in some form of productionized environment, evaluated the usability of the dashboards through qualitative approaches that involved surveys and interviews with learners. A further 12% of the studies created dashboard prototypes and conducted a qualitative investigation into their usability, while a same proportion developed prototypes, but did not evaluate them. A quarter of the studies performed a qualitative investigation into the effectiveness of the dashboards ability to impact student outcomes. These studies concluded that their LADs exhibited a positive impact on student outcomes. A common feature across half of the LADs which demonstrated a positive student outcome was they possessed predictive analytics capabilities. They all depicted information for each student in relation to where they were situated in respect to their peers across various metrics, and one of the dashboards implemented prescriptive features.

Our analysis indicates that 59% of the LADs have used six or more different colors on their display, potentially contributing towards information overload and miscommunication of insights. 29% used between four and six colors, while the remaining 12% employed up to 3 colors only.

Technology is an important aspect through which LADs ought to be considered. The chosen technology determines the range of capabilities of LADs and the agility of the projects. Where reported, Table 2 indicates that the chosen tools for implementing LADs have so far mostly been web application frameworks which carry with them a requirement of a high level of technical expertise. Usage of off-the-shelf commercial dashboarding products which do not require a high level of technical and programming expertise appear not to be a chosen medium yet. Both web application frameworks and off-the-shelf dashboarding products generally possess very limited advanced analytics capabilities, which would then require additional technologies to overcome this limitation. The exceptions being, Shiny (R) and Django (Python), where both technologies have access to a large ecosystem of analytics capabilities.

**Table 2:** Dashboard technologies and size of study cohorts

Study	Technology	Programming/expertise	Cohort size
Bodily et al., 2018	N/A		180
Chen et al., 2019	N/A		–
Aljohani et al., 2019	ASP MVC4, HTML5, jQuery and Highcharts JavaScript	High	86
Ulfa et al., 2019	N/A		67
Majumdar et al., 2019	N/A		–
He et al., 2019	HTML5, JavaScript and Echarts	High	327
Naranjo et al., 2019	Vue.js, HTML, CSS	High	64
Baneres et al., 2019	Web application	High	247
Gras et al., 2020	N/A		127
Karaoglan Yilmaz & Yilmaz, 2020	LMS messaging tool	Low	81
Fleur et al., 2020	Django	High	79
Chatti et al., 2020	Google charts and C3.js	High	414
Kia et al., 2020	JavaScript, D3.js	High	449
Owatari et al., 2020	Web application	High	108
Han et al., 2021	Web application	High	88
Kokoç & Altun, 2021	Google visualization API and AJAX API	High	126
Valle et al., 2021	R and Shiny	High	179

## Proposed learning analytics dashboard

**Fig. 3:**
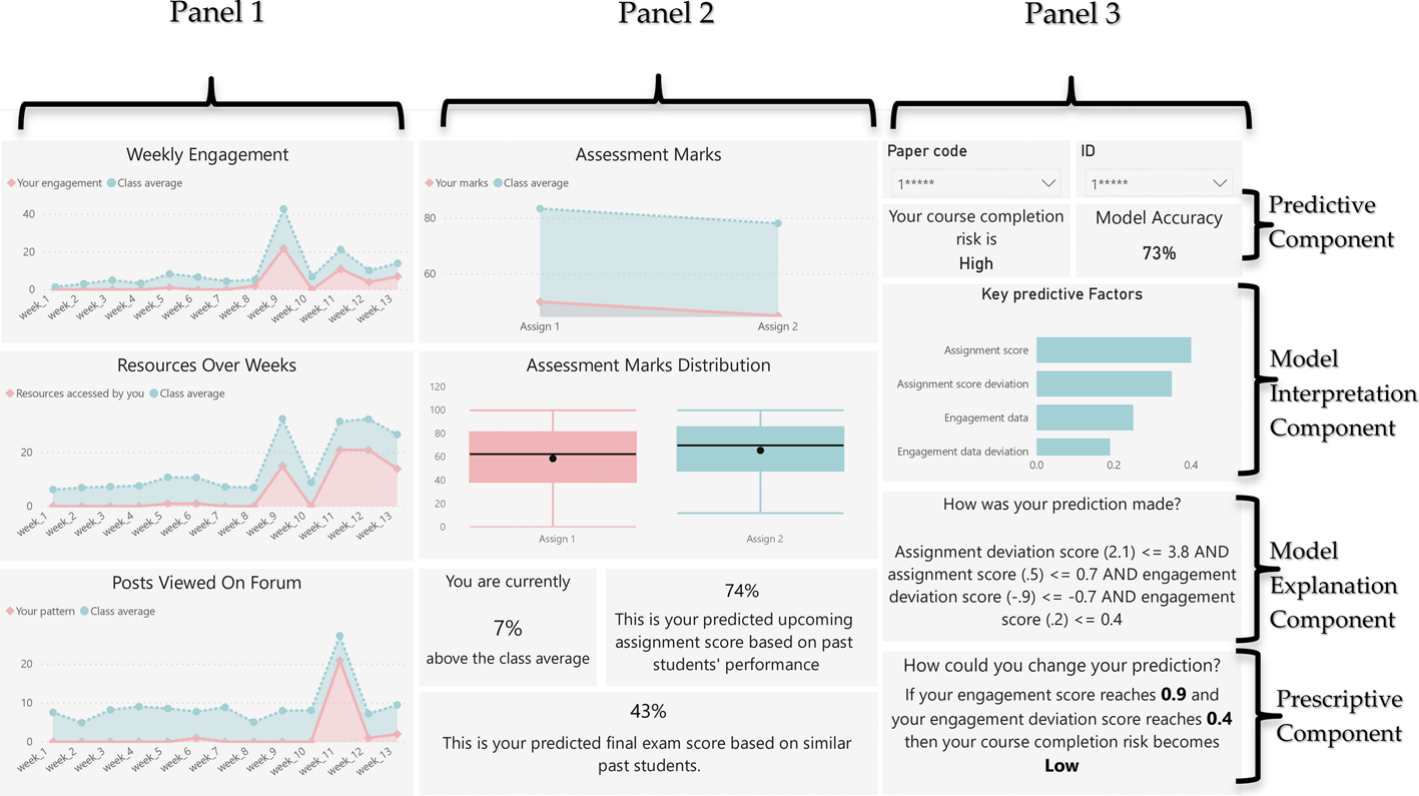
Learning analytics dashboard designed for students

The previous section reviewed dashboards and highlighted their key themes, analytic capabilities and reported efficacies. Drawing on contributions from these studies as well as the strengths and weaknesses of various dashboarding approaches, we propose our learner-facing dashboard design (shown in Fig. 3). The proposed dashboard attempts to integrate all levels of analytics capabilities missing in reviewed dashboards. The proposed dashboard has descriptive, predictive as well as prescriptive components built into it. To the best of our knowledge, this dashboard is the first of its kind to embed data-driven prescriptive capabilities involving counterfactuals into its display. In addition, our dashboard possesses a high degree of transparency and communicates to the learners how reliable the predictive models are; what the key factors are that drive the predictions, as well as the conversion of a black-box predictive model into a glass-box, human interpretable model for the learners so that they can understand how their prediction is being derived.

### Analytics layers

The proposed dashboard distributes the descriptive, predictive and prescriptive components across three panels seen in Fig. 3. The first panel highlights student engagement levels. This panel contains only descriptive analytics components and compares the learner’s engagements versus that of the cohort’ average. Engagement includes weekly login counts into the virtual learning environment, number of learning resources accessed, and total forum posts created as a measure of communication exchange levels.

The second panel displays information regarding a learner’s academic performance. This panel has both descriptive and predictive analytics components. The descriptive component in the top half, displays the snapshot of a learners’ assignment grades, quizzes and tests. The learner’s data is contrasted again with that of the cohort. The student can rapidly see their deviation from the class mean and can also inspect in greater detail how far their score deviates from their peers by viewing the overall class distribution through the box-and-whisker plots. The dashboard’s predictive component begins in the lower half of the second panel. This component provides a student with estimates of what scores they are likely to achieve in the upcoming assignment and their final exam based on the learners who have exhibited similar learning attributes in the past.

However, the key predictive analytics component and the novel prescriptive analytics features are found in the third panel. In this panel, an overall prediction is made regarding the learner’s estimated risk profile for meeting the course’s learning outcomes. Given the importance of this model, we emphasize key aspects of its nature that were missing in previous studies. The dashboard communicates the accuracy of the underlying model to the learner, and provides interpretability to the user in terms of what factors are deemed important to the model at a high-level when it makes a prediction. In addition, the dashboard contains an explainability component which communicates to the learner how the model has arrived at a given prediction for their individual case with the student’s specific input values. The model reasoning provides the learners with a suggestion of what they can alter in their learning behavior in order to alter their outcomes.

The above model transparency capability is further built upon and expanded by the dashboard’s prescriptive analytics features which incorporate counterfactuals. The counterfactuals indicate to the learner what specific factors together with minimal changes to their values, would produce different, and more positive predictive outcomes. The counterfactuals make some plausible assumptions about the existence of causal links in the underlying data, and based on this, generate automated advice to learners about how to maximize their learning outcomes.

From an aesthetic point of view, the dashboard attempts to minimize the use of color and renders the display in three hues, thus minimizing the risk of information overload. Additionally, the dashboard uses a neutral pastel palate to further reduce negative effects that colors can have, while attempting to maximize the data-to-ink ratio.

From a functional point of view, the proposed dashboard provides comprehensive analytics capabilities that are not found in existing LADs and demonstrates the state-of-the-art in terms of incorporating these functionalities. However, the dashboard is currently in a pilot stage at a tertiary institution with students from across 20 classes actively trialing the tool and evaluating it for usability; therefore, data on its effects on outcomes is not yet available.

### Dashboard design details

The underlying data for the dashboard originated from Moodle, an open-source learning management system which provides the virtual learning platform for e-learning at the institution. The dashboard was implemented following a client server architecture. On the client-side the Power BI^2^ tool was used to develop the web-based dashboard application. Meanwhile, on the server-side Python^3^ was used for both analytics and for the extract, transform, load phases.^4^

We used a mixture of Python’s scikit-learn library and the CatBoost (Dorogush et al., 2018) classification algorithm for generating student outcome predictions. The underlying features used to make the predictions were engagement deviation score, engagement rolling average score from the Moodle, assignment rolling average score, assignment deviation score, and previous grade from the student management system, and demographic information such as age, English equivalent test, and highest school qualification. Models were trained on a dataset comprising 4000 students. Hold-out method was used and the final accuracy from all the test datasets was displayed to the learners as a measure of confidence in the reliability of the underlying model. The dataset was divided into weeks and were used for the prediction analysis. Moreover, the data was used in a cumulative fashion for making predictions. For example, when making predictions for week 2 the students’ data from week 1 were taken into consideration as well. The reason being that prediction accuracy improves as more data becomes available in upcoming weeks. Prediction accuracy at early stages is important so that timely interventions can be made to help students.

Model interpretability was implemented used feature importance analysis which depicts the relative contribution and importance of each variable towards making predictions. This was also complemented with the use of anchors^5^ (Ribeiro et al., 2018). Anchors have recently devised as an approach for making black-box models interpretable. Anchors create proxy models which mimic the behavior of the underlying black-box model but present themselves to the user as a glass-box model. Proxy models are approximations of the real model and they present themselves as succinct human-readable decision rules.

In order to realize prescriptive capabilities, we used data-driven counterfactuals (Wachter et al., 2017) to suggest to students how an adjustment in certain behavioral learning patterns would result in a more positive prediction. For example, the counterfactual may suggest to a learner that an increase in their next assignment mark by a specific amount would change their classification from high-risk to low-risk. Such data-driven counterfactuals are based on correlation and do not guarantee causal links; however, in many cases when features are judiciously selected some degree of potential causality can safely be assumed. We use the Python counterfactual library^6^ (Mothilal et al., 2020) to generate the prescriptive analytics on the dashboards. The advantage with this tool is that the outputs are once again in a rule-based format and easy to comprehend. Additionally, the prescriptive suggestions represent a minimum shift in the values of key features that would need to take place in order to achieve a different outcome to what is currently predicted.

## Discussion

Our study reveals that learner-facing LADs are steadily gaining popularity (Fig. 2), while it is reasonable to assume that numerous others may have been deployed but remain unpublished. While the value of LADs are recognized by education providers, we find from literature that many of the published dashboards are only in their prototype phases, and only few in the pilot implementation stages. This is also in agreement with findings from other studies (Chen et al., 2019; Karaoglan Yilmaz & Yilmaz, 2020). A speculative link could be argued between the low deployment rates of LADs covered in this study and the underlying technology choices taken as seen in Table 2. The technologies used in the studies are heavy-duty in respect to design, development and maintenance of LADs, requiring significant resource investments and agility. As discussed, higher education organizations are short on both of the latter requirements at present which are therefore possibly contributing factors. Given that the reviewed LADs mostly used only descriptive analytics, it could be argued that off-the-shelf commercial dashboarding software would have delivered the same functionalities for a vastly reduced effort, with higher prospects of productionization.

Given the significant resources required to operationalize LADs, our study has revealed that there is paucity of evidence on their effectiveness to affect learner outcomes. This is again supported by Fig. 2 which suggests that learner-facing LADs are a relatively new and emerging technology in the LA space and so comprehensive and conclusive meta-study research into their effects has simply not yet taken place. We also see in Table 2 that most of the past LAD papers involved relatively small study cohorts to support conclusive findings, with the median being 126 subjects. Larger studies involving several hundred subjects are emerging, and more will be needed in future in order to answer this question concretely. Encouragingly, our research did find that about a quarter of the studies concluded that their LADs produced positive impacts on student outcomes. However, the number of studies were too small to determine which types of visualizations or dashboard features directly contributed towards positive impacts on learner outcomes. Further still, it is unclear if any effects could be attributed to dashboards themselves, or to the associated human interventions.

Given the ubiquity of machine learning now, it is a little surprising that predictive modelling has not featured in a larger percentage of reviewed LADs. A possible hypothesis could be that stringent ethics requirements and risk-averse positions taken by Research Ethics Committees may be playing a role. Some of the research using predictive analytics could also be encountering obstacles due to emerging legal requirements that predictive modelling is made completely transparent, interpretable and the predictions explainable to those affected, thus lifting much higher the barrier to entry for those seeking to leverage machine learning.

Undoubtedly though, data-driven prescriptive analytics represents the next frontier of LAD development. This is the most sophisticated level of analytics with the ability to offer learners evidence-based concrete suggestions or recommendations about what adjustments in learning behaviors would most likely result in positive outcomes (Aljohani et al., 2019; Lu et al., 2018).

### Future directions

Our final research question considers future directions of LA dashboarding and inquires into how some of the existing weaknesses can be addressed. We find that personalization of learning, which could be referred to as “precision learning” is the future, and there is a role for LADs in supporting this through embedding of recommendation-like features which suggest next steps to learners for maximizing outcomes. In addition, LADs can take on greater roles in early intervention responses to learners identified as being at-risk. Integrating automated interventions within the dashboards and evaluating their effectiveness will be one of the future research directions. The focus on personalized learning and early interventions as an area of needed focus is also supported by (Gras et al., 2020; Han et al., 2021) while closing the loop and ensuring that the insights generated by LA systems, or dashboards, is actionable and not just interesting, is emphasized by (Baneres et al., 2019; Chen et al., 2019).

Maisarah et al. (2020) noted in their broad survey of LADs the importance of embedding customization capabilities within dashboards in order to make them user-friendly and thus promote long-term usage of the dashboards. Future research will focus on developing technologies that possess these capabilities and are able to seamlessly integrate with native platforms used by institutions for their existing Virtual Learning Environments. Leitner et al. (2019) also mention the utility of embedding analytics within dashboards themselves in order to directly gather information on learner usage patterns of the dashboards themselves in order to optimize them in subsequent iterations of development.

Lastly, Sedrakyan et al. (2020) go further and ambitiously suggest integrating data from activities in the learning-process which may not be directly linked with the institutional learning environments. They propose data acquisition from multi-modal sources such as biofeedback from various wearable sensors, audio/video streams and using them to augment LADs. Thus, scalability in processing capabilities of live data streams originating from wearable sensors would form yet another requirement of future work for LADs.

### Study limitations

We acknowledge that the search time-window of 2018 to 2021 is constrained, and that data from 2021 is partially collected which constitutes a limitation of this study. Data on the usability of the proposed dashboard and its effects on student outcomes are being collected. A further limitation of this study is that these data cannot yet be presented, neither can this tool be made available publicly for trial purposes at this point in time due to software licensing constraints.

## Conclusion

Learning Analytics dashboards (LADs) are becoming increasingly commonplace within the educational sector with the aims of improving the quality of the learning experience and thereby maximizing learner outcomes. Our study focused on identifying challenges associated with LAD projects as well as analyzing characteristics of recent advances in LADs. We comprehensively surveyed existing LADs and analyzed them through the prism of the sophistication of insights they deliver and ways in which they help learners make informed decisions about making adjustments to their learning habits. Finally, in considering the strengths and weaknesses of existing LADs, we propose a dashboard currently being deployed for trials at a tertiary institution that attempts to address some of the gaps we found in literature. Our research findings have both theoretical and practical implications.

### Theoretical implications

We have added to the body of knowledge surrounding what we know to be challenges in operationalizing Learner Analytics (LA) projects. We refined these challenges to LAD projects and have identified the lack of agility in higher education institutions as one of the key pressure points. Our work has confirmed that learner-facing LADs are on the rise within higher education institutions, but significant gaps in understanding and quantifying the effectiveness of LADs exists. In particular, uncertainty exists about which components within LADs are more effective at improving learning outcomes. We find that predictive modeling functionalities are not used in majority of cases within the reviewed LADs, and examples of interpretability of the models and the ability to explain their predictions to the learners do not yet exist in published studies. Additionally, our study reveals the absence of data-driven prescriptive analytics which, with other gaps, highlights numerous worthwhile avenues for future studies to pursue.

### Practical implications

A key practical implication of this study is a demonstration of how a sophisticated LAD can be developed which integrates all forms of analytics: descriptive, predictive and prescriptive. We have demonstrated how interpretability of predictive models can be made available to the learners and critically, how the specific predictions for a given learner can be explained to them. This will establish trust with the users through transparency of moving beyond black-box predictive models, and in the process satisfy emerging regulatory requirements. Additionally, we have demonstrated how automated and data-driven prescriptive analytics can be leveraged within LADs. Our research also points the analytics practitioners towards recently developed technologies which more than ever, make these capabilities accessible to the wider audience.

## Data Availability

Not applicable.

## Abbreviations

AA: Academic Analytics
EDM: Educational Data Mining
LA: Learning analytics
LADs: Learning analytics dashboard(s)
RQ(s): Research question(s)
VLE: Virtual Learning Environment

## Footnotes

1 The General Data Protection Regulation (GDPR) is an example of a regulation requiring a “right to explanation” in respect to predictive models.

2 Power BI is a commercial software package owned by Microsoft. Effective at building dashboards, but at present lacks capabilities for predictive and prescriptive modelling, as well as model interpretability.

3 Python is a general-purpose programming language with a rich set of capabilities for implementing all required analytics; however, it lacks easy-to-use capabilities for building front-end dashboards.

4 Both Power BI and Python can effectively be substituted for R and it’s R Shiny technology for constructing dashboards and developing the underlying analytics functionalities.

5 Our implementation of anchors used the anchor-exp library https://pypi.org/project/anchor-exp/

6 Dice-ml https://pypi.org/project/dice-ml/
